# Effectiveness, acceptability and feasibility of an Internet-delivered cognitive behavioral pain management program in a routine online therapy clinic in Canada

**DOI:** 10.1080/24740527.2018.1442675

**Published:** 2018-03-21

**Authors:** Heather D. Hadjistavropoulos, Luke H. Schneider, Thomas Hadjistavropoulos, Nickolai Titov, Blake F. Dear

**Affiliations:** a Department of Psychology, University of Regina, Regina, SK, Canada; b MindSpot Clinic, Australian Hearing Hub Building, eCentreClinic, Department of Psychology, Macquarie University, Sydney, NSW, Australia; c eCentreClinic, Department of Psychology, Macquarie University, Sydney, NSW, Australia

**Keywords:** chronic pain, anxiety, depression, cognitive behavioral therapy, Internet-delivered

## Abstract

**Background:**

Access to face-to-face cognitive behavioral pain management programs is very limited. Internet-delivered cognitive behavioral pain management has potential to improve client access to care but is not readily available in Canada.

**Aims:**

The present study explored the effectiveness, acceptability, and feasibility of a previously validated Internet-delivered cognitive behavioral pain management course, the Pain Course, when offered in a publicly funded provincial Online Therapy Clinic. The five-lesson course was delivered over 8 weeks and was accompanied by brief weekly contact from a coach via weekly telephone calls and secure online messages.

**Methods:**

A single-group open trial design (ISRCTN15509834) was employed (*n* = 55). Effectiveness was assessed by examining symptom measures at pretreatment, posttreatment, and 3-month follow-up. Completion rates and satisfaction ratings were used to examine acceptability. Feasibility was assessed by examining time required for service delivery.

**Results:**

Results were highly comparable to past studies of the Pain Course showing improvements on primary measures of disability (Cohen’s *d* = 0.45; 18% reduction), depression (Cohen’s *d* = 0.85; 36% reduction), and anxiety (Cohen’s *d* = 0.52; 32% reduction) at posttreatment that were maintained at follow-up. Completion rates (76%) and course satisfaction ratings (85% would recommend course) were high. Coach time per week was estimated as M = 12.67 (SD = 6.53) min.

**Conclusions:**

The findings add to existing literature on the Pain Course demonstrating for the first time the effectiveness, acceptability, and feasibility of Internet-delivered cognitive behavioral pain management programs for adults with chronic pain in a routine online therapy clinic.

## RÉSUMÉ

**Contexte**: L’accès aux programmes de prise en charge cognitivo-comportementale de la douleur en mode présentiel est très limité. La prise en charge cognitivo-comportementale de la douleur par Internet a le potentiel d’améliorer l’accès aux soins, mais elle n’est pas facilement disponible au Canada.

**Buts**: Cette étude portait sur l’efficacité, l’acceptatbilité et la faisabilité d’un cours de prise en charge cognitivo-comportementale de la douleur par Internet précédemment validé, le *Pain Course*, offert par une clinique de traiement en ligne financée par des fonds publics. Ce cours en cinq lessons, qui s’est déroulé sur une période de huit semaines, s’accompagnait d’un bref contact hebdomadaire avec un coach par le biais d’appels téléphoniques et de messages en ligne

**Méthodes**: Un devis d’essai ouvert à un seul groupe (ISRCTN15509834) a été utilisé (n = 55). L’efficacicté a été évaluée par la mesure des symptômes avant le traitement, après le traitement et lors d’un suivi trois mois aprés le cours. Les taux d’achèvement et de satisfaction ont été utilisés pour évaluer l’acceptabilité. La faisaibilité a été évaluée en mesurant le temps requis pour la prestation du service.

**Résultats**: Les résultats étaient fortement comparables aux études antérieures sur le *Pain Course* et ont démontré des améliorations dans les principales mesures de l’incapacité (*d* de Cohen = 0,45; réduction de 18 %), de la dépression (*d* de Cohen = 0,85; réduction de 36 %), et de l’anxieté (*d* de Cohen = 0,52; réduction de 32 %) après le traitement, et qui étaient toujours présentes lors du suivi. Les taux d’achètevement (76 %) et de satisfaction à l’égard du cours (85 % des personnes recommenderaient le cours) étaient élevés. Le temps d’encadrement a été estimé à *M* = 12,67 (écart type = 6,53) minutes par semaine.

**Conclusions**: Ces résultats enrichissent la littérature existante sur le *Pain Couse* en démontrant pour la première fois l’efficacité, l’acceptabilité et la faisabilité de programmes de prise en charge cognitivo-comportementale de la douleur par Internet destinés aux adultes souffrant de douleur chronique dans le cadre d’une clinique de traitement de routine en ligne.

## Introduction

Chronic pain is prevalent, disabling, costly, and undertreated.^1–4^ Psychological distress is present in many individuals with chronic pain, and cognitive behavioral pain management programs are recognized as beneficial for alleviating suffering related to chronic pain.^5^ Access to such treatment programs, however, is very limited, in terms both of availability and affordability.^6^ Delivery of cognitive behavioral pain management programs via the Internet has the potential to improve the accessibility of services for individuals who have chronic pain. These programs use structured online lessons, released gradually over time, to provide individuals with the same information and skills as those taught in face-to-face pain management programs. These programs can be either self-guided or delivered with brief contact from a health care professional or trained coach via secure e-mail-type messaging or telephone.^7^ The primary purpose of the health care professional or coach is to encourage client completion of materials and answer questions regarding program materials as needed.^8^ A survey of people with chronic pain suggests that most individuals perceive these programs as valuable.^9^

One program that shows particular promise is the Pain Course.^8,10,11^ This course simultaneously addresses pain and disability but also depression and anxiety, which are highly prevalent among people with chronic pain. The Pain Course is effective for diverse pain-related conditions, and the outcomes of the course parallel those found for face-to-face pain management programs.^8,10,11^ Of interest, the outcomes of this course are similar whether delivered with weekly clinician contact, client-directed optional contact with a clinician,^8^ or support from a nonclinician.^12^ Strong outcomes, regardless of the type of support, have been attributed to use of prescreening to ensure that clients are appropriate for the course (e.g., are not at high risk of suicide or suffering from severe mental health problems, facilitate client understanding of the course) and to the high-quality treatment materials, which reduce the need for clinician support.^11^

Though the results are very encouraging, there has been limited research on the Pain Course outside of research trials in clinical settings where clients are not recruited but instead seen on a routine basis. In clinical as compared to research settings, clients can present with greater severity and comorbidity and there can be dilution of treatment fidelity.^13^ Therefore, it cannot be assumed that Internet-delivered pain management programs will be equally effective or acceptable when employed in busy clinical settings. Implementation trials are important for establishing the generalizability of interventions especially prior to broad-scale dissemination. Implementation trials also offer the opportunity to understand the feasibility of offering the intervention in terms of resources required for delivery.^14^

The present study used an open trial design to extend the available literature on the Pain Course within Canada, and provide important information for further implementation efforts, by examining the effectiveness, acceptability, and feasibility of delivering the Pain Course within an online clinical setting. More specifically, the Pain Course was offered within an online therapy clinic that routinely offers Internet-delivered cognitive behavioral therapy to people who suffer from depression and or anxiety.^15^ In order to reduce the costs associated with implementation, the Pain Course was delivered with assistance of a coach rather than a health care professional because past research suggests that this approach can be as effective as therapist assistance but is less costly to implement.^7^ Effectiveness was assessed by collecting symptom measures at pretreatment, posttreatment, and 3-month follow-up. Completion rates and satisfaction ratings were used to examine acceptability. Feasibility was assessed by examining personnel time required for delivery of support. Using an open trial design, it was hypothesized that (1) clients would report significant improvements on measures of pain severity, disability, depression, anxiety, fear of movement, self-efficacy, and chronic pain acceptance from pretreatment to posttreatment and these improvements would be maintained at 3-month follow-up and (2) there would be high completion rates (above 80%) and high satisfaction rates. No hypotheses were put forth regarding time required to deliver the course.

## Methods

### Context

This trial took place in Saskatchewan, Canada, a province with a population estimate of ~1.1 million.^16^ The Online Therapy Unit receives financial support from the provincial government to offer Internet-delivered cognitive behavioral therapy. This funding is designed to address concerns that many individuals report that their mental health needs are unmet or only partially met.^17^

### Clients

All clients applied for the Pain Course through the Online Therapy Unit (www.onlinetherapyuser.ca). All clients who completed the online eligibility screening process between June 17, 2015, and September 20, 2016, were included in the trial. Clients learned about the online treatment via medical professionals (27%; *n* = 15), mental health professionals (40%; *n* = 22), word-of-mouth (14%; *n* = 8), online searches and e-mail announcements (14%; *n* = 8), media (2%; *n* = 1), and printed posters/cards (2%; *n* = 1).

**Figure 1.**
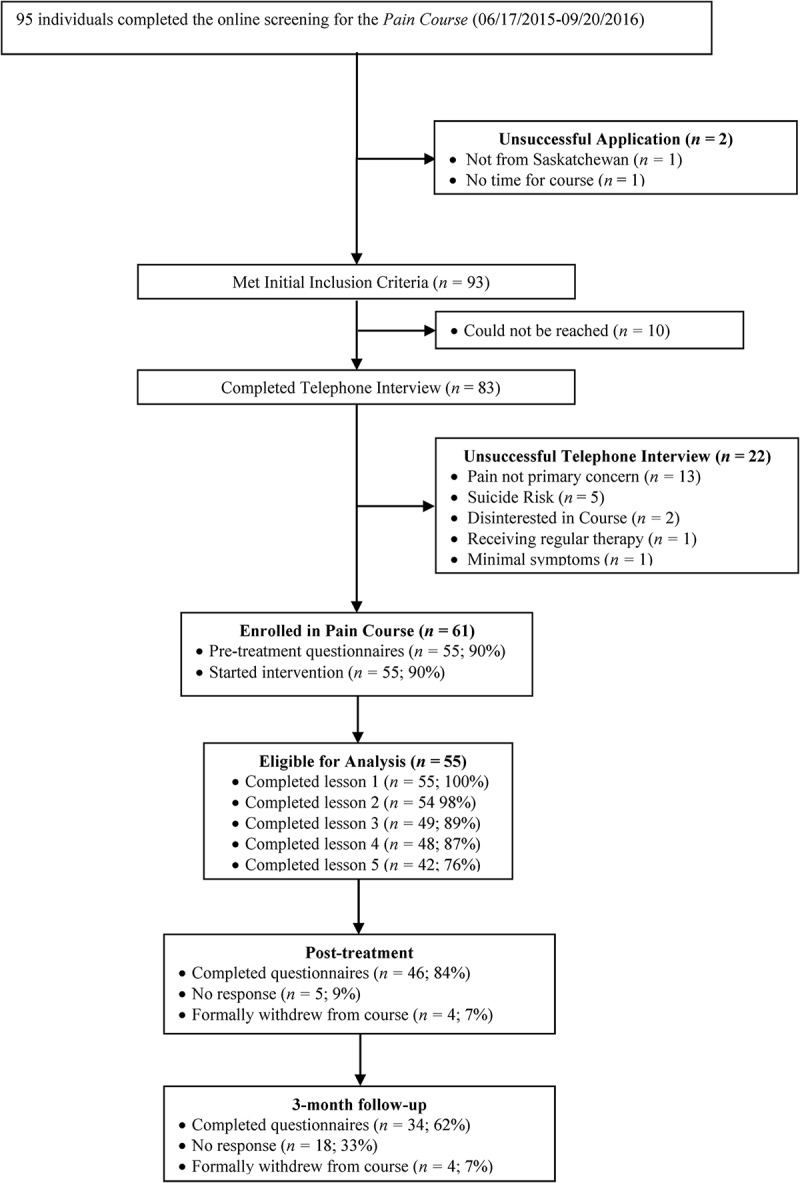
Participant flowchart.

A total of 93 individuals applied for the Pain Course, of whom 55 met inclusion criteria and completed pretreatment questionnaires. Participants reported pain as a result of various injuries, medical treatments, as well as a broad range of other significant health conditions (e.g., fibromyalgia, multiple sclerosis, degenerative disease). Demographic and pain characteristics of the study sample are presented in Table 1. Consistent with previous use of the course,^8,10,11^ clients were eligible for the current study if during the online screening and subsequent telephone interview they reported that they were (1) residing in the province, (2) 18 years of age or older, (3) experiencing pain for 3 months or more with past contact with a physician about their pain, (4) willing to provide a physician as an emergency contact, (5) concerned about low mood or worry but not about high risk of suicide, (6) comfortable using computers and the Internet, (7) willing to dedicate time each week to the course completion, and (8) not receiving regular face-to-face therapy. See Figure 1.

**Table 1.** Patient characteristics and program engagement.

	*n*	%
Age		
Mean (SD)	44.07 (14.09)	—
Range	21–70	—
Gender		
Male	16	29
Female	39	71
Marital status		
Single/never married	12	22
Married/common law	34	62
Separated/divorced/widowed	7	13
Undisclosed	2	3
Education		
Less than high school	1	2
High school diploma	11	20
Post high school certificate/diploma	13	24
University education	30	54
Employment status		
Employed part-time/full-time	21	38
Unemployed	5	9
Homemaker	2	4
Student	3	5
Retired	6	11
Short-term disability	8	15
Long-term disability	10	18
Ethnicity		
Caucasian	49	89
Indigenous	2	4
Other	3	5
Undisclosed	1	2
Location		
Large city (over 200,000)	25	45
Small city	14	26
Small rural location	16	29
Duration of pain symptoms (years)		
Mean (SD)	6.00 (7.53)	—
Range	0.25–42.00	—
Pain location		
Upper back/middle back/lower back	43	78
Hip/pelvis/leg/foot	36	65
Shoulder/arm/hand	25	45
Head/face	19	35
Other	13	24
Average number of pain sites (SD)	4.51 (3.06)	—
Prescription medication		
Pain	44	88
Mental health	29	53
Prescription medications reported^a^		
Strong opioid analgesics	18	33
Weak opioid analgesics	11	20
Anticonvulsants	16	29
Nonsteroidal anti-inflammatories	12	22
Muscle relaxants	4	7
Benzodiazepines	5	9
Anxiolytics and antidepressants	37	67
Other pain or mental health medications	13	24
Mean number of prescription medications reported (SD)	2.11 (1.55)	—
Mental health characteristics		
Infrequent use of some form of mental health treatment	25	45
Pretreatment GAD-7 ≥ 10	23	42
Pretreatment PHQ-9 ≥ 10	42	76
Program engagement		
Completion of four lessons	48	87
Completion of five lessons	42	76
Completion of posttreatment questionnaires	46	84
Completion of 3-month follow-up questionnaires	34	62
Mean number of log-ins (SD)	22.18 (14.97)	—
Mean days between first and last log-in (SD)	86.51 (49.88)	—
Mean number of phone calls with therapist (SD)	5.40 (2.05)	—
Mean written messages sent to therapist (SD)	2.36 (2.76)	—
Mean written messages received from therapist (SD)	4.60 (2.05)	—

^a^Only prescription medications for pain, a pain-related condition, anxiety, or depression are reported. Strong opioids: buprenorphine, fentanyl, hydromorphone, methadone, morphine, oxycodone; weak opioids: codeine, tramadol, tapentadol; anxiolytics, and antidepressants: beta-blockers, selective serotonin reuptake inhibitors, norepinephrine and serotonin–norepinephrine reuptake inhibitors, tricyclics, and tetracyclics; other psychotropic or pain medications: corticosteroids, antispasmodics, serotonin agonists, dopamine agonists, antipsychotics, and psychostimulants.

GAD-7 = Generalized Anxiety Disorder 7-Item; PHQ-9 = Patient Health Questionnaire 9-Item.

Clients visited the online therapy website (www.onlinetherapyuser.ca) where they learned of the Pain Course. After providing informed consent, they were directed to a preliminary online screening questionnaire that assessed eligibility criteria. This was followed by a detailed telephone screening (taking ~30 min) completed by a clinician in the Online Therapy Unit. The telephone call was conducted to ensure that clients met the study inclusion criteria, were not at high risk of suicide, and also gave clinicians an opportunity to discuss the requirements of the Pain Course and the consent form with clients. Following telephone screening, eligible clients were immediately enrolled in the intervention. Clients received no remuneration for their participation.

### Design and measures

This study involved a longitudinal single-group open-trial design to assess the effectiveness, acceptability, and feasibility of the Pain Course when delivered with support of a coach within the Online Therapy Unit. This study received institutional research ethics approval and was registered with the Current Controlled Trials Register (ISRCTN15509834) prior to commencement. Clients completed standardized measures at pretreatment and posttreatment and 3-month follow-up. All measures were completed online.

### Primary measures

#### Roland Morris Disability Questionnaire

The Roland Morris Disability Questionnaire (RMDQ) consists of 24 items rated on a yes/no scale and assesses client ability to engage in various day-to-day activities.^18^ Consistent with past research,^11^ the word *back pain* was changed to *pain* so that the scale would apply to a broad range of chronic pain conditions. The validity of this modified version has been established.^19^ As previously identified by Dear at al.,^8^ a total score ≥ 14 on the RMDQ was used to classify scores within the clinical range. In the present study, Cronbach’s α ranged from 0.85 to 0.91 across administrations.

#### Generalized Anxiety Disorder 7-item

The Generalized Anxiety Disorder 7-Item (GAD-7) measures generalized anxiety using seven items rated 0 (*not at all*) to 3 (*nearly every day*). A score of 8 or greater identifies individuals likely to meet diagnostic criteria for generalized anxiety disorder.^20^ The GAD-7 possesses excellent psychometric properties.^21^ In the present study, Chronbach’s α ranged from 0.90 to 0.94.

#### Patient Health Questionnaire 9-item

The Patient Health Questionnaire 9-Item (PHQ-9) consists of nine items assessing depression, including suicidality. Items are rated from 0 (*not at all*) to 3 (*nearly every day*).^22^ Past research has identified that a score of 10 or greater is associated with a likely diagnosis of major depression.^23^ The PHQ-9 has strong psychometric properties.^22,24,25^ In this study, Cronbach’s α ranged from 0.84 to 0.87.

### Secondary measures

#### The Brief Pain Inventory

The Brief Pain Inventory (BPI) is a measure that is designed to assess the location, severity, and interference of pain on daily functions.^26^ Only the four BPI pain severity items were used in the current study. These items asked individuals to rate the intensity of their current pain, average pain, least pain in the past 24 h, and worst pain in the last 24 h using a scale that ranged from 0 (*no pain*) to 10 (*pain as bad as you can imagine*). Consistent with BPI scoring practices,^26^ the BPI pain severity items were combined to create a mean composite score. Psychometric studies for the BPI support the measure^26^ and Cronbach’s α in the current study ranged from 0.88 to 0.92.

#### Pain Self-Efficacy Questionnaire

The Pain Self-Efficacy Questionnaire (PSEQ) is a brief measure consisting of ten items that assess client beliefs about their ability to undertake a number of daily tasks regardless of pain.^27^ Ratings are made on a seven-point scale, with higher scores indicating a greater level of pain self-efficacy. The PSEQ has good psychometric properties.^27^ Cronbach’s α in the current study ranged from 0.90 to 0.96.

#### TAMPA Scale of Kinesiophobia

The TAMPA Scale of Kinesiophobia (TSK) is composed of 17 statements measuring fear of movement and re-injury using a four-point scale.^28^ The TSK has strong reliability^29^ and validity.^28,30^ Cronbach’s α in this study ranged from 0.81 to 0.89.

#### Chronic Pain Acceptance Questionnaire 8-item

The Chronic Pain Acceptance Questionnaire 8-Item (CPAQ-8) is an eight-item measure that measures acceptance of chronic pain, with higher scores indicating greater willingness to experience and greater acceptance of pain.^31^ The CPAQ-8 has strong psychometric properties.^31^ Cronbach’s α in this study ranged from 0.85 to 0.87.

### Program acceptability

To assess program acceptability, we examined the number of Pain Course lessons clients completed, the number of times clients accessed the program, the number of online messages exchanged with the coach, and the number of telephone calls. Furthermore, at posttreatment, consistent with other Internet intervention research,^11,32,33^ clients reported whether they were satisfied with the course, would recommend the course to a friend, and thought that the course was worth their time.

### Resources

To assess resources required to deliver the course, the assigned coach tracked minutes spent each week providing support via telephone or secure messages.

### Treatment program

The Pain Course was developed and is owned by the eCentreClinic (www.ecentreclinic.org) at Macquarie University and was licensed (at no cost) by the Online Therapy Unit.^11^ It consists of five online lessons presented in a slide show format with text and images. Lessons of the Pain Course focus on (1) psychoeducation regarding chronic pain, depression, and anxiety, including the cognitive behavioral model and the relationship between physical symptoms, thoughts, and behaviors; (2) thought monitoring and challenging related to pain, depression, and anxiety; (3) controlled breathing and pleasant activity scheduling to manage underarousal and overarousal; (4) activity pacing and graded exposure to manage behaviors associated with pain, depression, and anxiety; and (5) relapse prevention including helping clients recognize signs of relapse and the importance of goal setting. The slide shows are delivered to clients sequentially over the course of 8 weeks, with clients given 1 week to work on lessons 1 and 3 and 2 weeks to work on lessons 2, 4, and 5. Lessons are complemented by lesson summaries that can be downloaded and retained by clients as well by recommended homework assignments that facilitate learning of different skills. Client stories are also shared to illustrate how past clients have used the skills to cope with chronic pain. In addition to core lessons, there are additional downloadable handouts on topics that are often of value to individuals who have chronic pain (i.e., sleep, working with health professionals, common methods for treating pain, suicide resources, problem solving, managing beliefs, attention, panic, pleasant activities, and assertiveness). To access all materials, clients were given a username and password to sign onto the intervention website. Minor adaptations were made to the Pain Course for the current study to reflect the Canadian context (e.g., Canadian statistics and language). See Dear et al.^11^ for complete details regarding the Pain Course.

### Support

Consistent with the standard delivery of the Pain Course,^11^ contact with clients occurred via secure messages and telephone. Support was provided by a coach who was a doctoral-level clinical psychology graduate student (L.S.) who had past graduate training in chronic pain and Internet-delivered therapy. The coach acted under the supervision of a registered doctoral psychologist. There is strong evidence that nonclinicians are able to provide guidance (e.g., students, minimally trained support staff) without compromising clinical outcomes or acceptability.^7,34,35^ Over the course of 8 weeks, the coach contacted clients via telephone. If the client could not be reached via telephone, a secure message was sent via the intervention website that conveyed information similar to that provided in the telephone check-in. In these calls, the coach summarized content, answered questions, reinforced completion of the course, and encouraged practice of skills. Additionally, the coach normalized challenges in learning skills. The coach did not introduce any new therapeutic skills and did not provide any therapeutic advice. In addition to the above contact, clients received standardized automated messages each week. These messages notified clients of upcoming material, encouraged the use of skills, and provided strategies to address barriers to skill use. The coach was encouraged to spend 10 to 15 min per client each week. It was possible, however, to spend more or less time depending on client response.

### Analyses

All analyses were conducted using Statistical Package for the Social Sciences version 23. The sample and outcome measures were first described using descriptive statistics (e.g., means, standard deviations, percentages). Changes in measures over time were examined using a generalized estimating equation (GEE). GEE analyses allow for examination in changes in measures over time, while also accounting for within-subject variance through the use of a working correlation model.^36^ The GEE model provides model coefficients representative of a change in the dependent variable, allowing for the calculation of the average percentage change from baseline to posttreatment and follow-up. For all GEE analyses, an exchangeable working correlation and robust error estimation were selected. Prior to analyses, the distribution of each dependent variable was examined to address skewness, and each GEE model specified either a normal or gamma with log link response scale. Following intention-to-treat principles, missing data were imputed using separate generalized linear models that utilized time effects and random intercepts.

To assist with interpretation of the results, a number of statistics were calculated based on the GEE analyses. First, for each outcome variable, we calculated (1) the average percentage change across time with 95% confidence intervals and (2) Cohen’s *d* effect sizes and associated 95% confidence intervals for the within-group effects based on the estimated marginal mean values derived from the GEE models.

Consistent with recommendations for reporting negative outcomes in Internet-delivered cognitive behaviour therapy (ICBT) trials^37^ and consistent with previous Pain Course trials,^8,11^ the number of clients reporting symptom deteriorations of 30% or greater and symptoms in clinical ranges at posttreatment are reported for the GAD-7, PHQ-9, and RMDQ. These analyses were designed to provide information on participants who demonstrated meaningful deterioration in symptoms throughout the course rather than nonsignificant fluctuations in scores (e.g., a change from 0 to 1). Descriptive statistics were used to examine completion rates (e.g., percentage of clients who started treatment who completed each of the main lessons), treatment satisfaction, and time required to deliver the Pain Course.

Exploratory analyses were used to assess the impact of amount of coach contact on program outcome. Pearson correlation coefficients were used to determine the relationship between coach contact time and residual change scores on primary outcome measures. Residual change scores were calculated with the formula *Z*_2_ − (*Z*_1_ * *R*_12_), such that a positive residual change score signified deterioration (i.e., time 2 is greater than time 1) and a negative change score signified improvement (e.g., time 2 score is less than time 1) from pre- to posttreatment. Residual change scores were used because they account for individual differences as well as multiple administrations of measures.^38^

## Results

### Baseline data, adherence, and attrition

Demographic characteristics are presented in Table 1 and pretreatment scores on primary and secondary measures are presented in Table 2. Details regarding participant flow are presented in Figure 1. Of the clients who began the course, 87% completed four of five lessons and 76% completed all five lessons. A majority of clients provided data at posttreatment (*n *= 46; 84%) and at 3-month follow-up (*n *= 34; 62%). Four clients formally withdrew from the course due to time constraints (*n* = 3) and a loss of Internet access (*n* = 1).

**Table 2.** Means, standard deviations, and effect sizes for the primary and secondary outcome measures.^a^

	Estimated marginal mean values			Percentage change from baseline^b^		Cohen’s *d* effect sizes from pretreatment	
	Pretreatment	Posttreatment	3-Month follow up	Posttreatment	3-Month follow up	Posttreatment	3-Month follow up
Primary outcomes							
GAD-7	9.33 (5.40)	6.36 (6.00)	6.49 (6.62)	32 (12 to 47)	30 (9 to 47)	0.52 (0.14 to 0.90)	0.47 (0.09 to 0.85)
PHQ-9	13.07 (5.48)	8.33 (5.67)	9.10 (6.83)	36 (24 to 47)	30 (15 to 43)	0.85 (0.46 to 1.24)	0.64 (0.26 to 1.02)
RMDQ	14.44 (4.93)	11.84 (6.48)	11.19 (6.41)	18 (6 to 30)	22 (11 to 34)	0.45 (0.07 to 0.83)	0.57 (0.19 to 0.95)
Secondary outcomes							
BPI-Severity	5.15 (1.74)	4.94 (1.95)	4.70 (1.75)	4 (−6 to 14)	9 (0 to 18)	0.11 (−0.26 to 0.49)	0.26 (−0.12 to 0.63)
CPAQ-8	18.91 (8.38)	25.16 (9.35)	26.42 (9.12)	25 (16 to 34)	28 (20 to 37)	0.70 (0.32 to 1.09)	0.86 (0.47 to 1.25)
PSEQ	24.18 (11.73)	34.20 (12.51)	40.48 (26.02)	29 (20 to 38)	40 (33 to 48)	0.83 (0.44 to 1.22)	0.81 (0.42 to 1.20)
TSK	41.15 (7.08)	37.20 (7.70)	39.21 (10.85)	10 (4 to 14)	5 (−3 to 11)	0.53 (0.15 to 0.91)	0.21 (−0.16 to 0.59)

^a^SDs and confidence intervals are shown in parentheses for means, percentage change, and effect sizes, respectively.

^b^The percentage change from baseline statistics are estimates of relative change derived from the GEE models conducted separately for each outcome.

GAD-7 = Generalized Anxiety Disorder 7-Item; PHQ-9 = Patient Health Questionnaire 9-Item; RMDQ = Roland Morris Disability Measure; BPI = Brief Pain Inventory; CPAQ-8 = Chronic Pain Acceptance Questionnaire 8-Item; PSEQ = Pain Self-Efficacy Questionnaire; TSK = TAMPA Scale of Kinesiophobia.

### Primary outcome measures

The means, standard deviations, percentage reductions, and Cohen’s *d* effect sizes for the primary measures (RMDQ, PHQ-9, and GAD-7) are presented in Table 2. The GEE analyses revealed significant time effects for these measures, including the GAD-7 (Wald’s χ^2^ = 19.89, *P* < 0.001), PHQ-9 (Wald’s χ^2^ = 42.59, *P* < 0.001), and RMDQ (Wald’s χ^2^ = 32.04, *P* < 0.001). Planned contrasts revealed statistically significant reductions from pre- to posttreatment on the GAD-7, PHQ-9, and RMDQ (*P*s < 0.001). There were no differences between posttreatment and follow-up scores on the primary measures (*P* range = 0.320–0.866), indicating maintenance of symptom reductions at follow-up.

### Secondary outcome measures

The GEE analyses revealed significant time effects for all secondary measures, including the BPI (Wald’s χ^2^ = 22.69, *P* < 0.001), CPAQ (Wald’s χ^2^ = 170.45, *P* < 0.001), PSEQ (Wald’s χ^2^ = 52.10, *P* < 0.001), and TSK (Wald’s χ^2^ = 20.50, *P* < 0.001). Planned contrasts revealed statistically significant reductions from pre- to posttreatment for the CPAQ, PSEQ, and TSK (*P* range <0.001–0.004). There were no statistically significant reductions from pre- to posttreatment for the BPI (*P* = 0.272); however, the reduction of scores from pretreatment to follow-up was statistically significant (*P* < 0.001). There were no statistically significant differences in scores from posttreatment to follow-up on the PSEQ and TSK (*P* range = 0.062–0.092), but a statistically significant difference was observed from posttreatment to follow-up for the CPAQ (*P* = 0.049), indicating further improvement at 3-month follow-up.

### Clinical significance

Percentage change as well as within-group effect sizes from the GEE models are shown in Table 2. Significant percentage improvements were observed for the primary measures from pre- to posttreatment (range 18%–36%), as well as on the CPAQ (25%) and PSEQ (29%). Notably, smaller percentage improvements from pre- to posttreatment were observed for the BPI (4%) and TSK (10%). Large within-group effect sizes (Cohen’s *d*) were observed for the PHQ-9 and PSEQ (*d* range = 0.83–0.85) and medium between-group effect sizes were observed for the GAD-7, CPAQ, and TSK (*d* range = 0.52–0.70). Small within-group effect sizes were observed for the BPI and RMDQ (*d* range = 0.11–0.45).

### Clinical deterioration

Two percent of clients (1/55) were classified as having deteriorated depression scores (e.g., increased depression score in the clinical range) and 5% of clients (3/55) were classified as having deteriorated anxiety scores at posttreatment. No clients were classified as having deteriorated disability scores at posttreatment.

### Treatment satisfaction

Of the clients who completed the posttreatment satisfaction measures, 73% (25/34) reported being either satisfied or very satisfied with the overall program and the quality of the course materials. The majority of clients also reported that they thought the course was worth their time (88%; 30/34) and that they would recommend it to a friend (85%; 29/34).

### Time spent and summary of contacts

On average, clients logged into the course 23.18 times (SD = 14.97). During the program, the mean number of online messages sent by clients to the coach was 2.36 (SD = 2.76) messages. The coach made an average of 6.95 (SD = 2.05) telephone calls per participant (this included both answered and unanswered calls) and sent an average of 4.60 (SD = 2.05) messages per participant. The mean total contact per client was 108.27 min (SD = 38.08). This time consisted of speaking with the client on the phone as well as leaving phone messages when clients did not answer the phone (M = 75.40; SD = 37.48) as well as reading and responding to client e-mails (M = 32.87; SD = 18.61). Exploratory analyses of contact time revealed a statistically significant positive linear relationship between coach contact and PHQ-9 change scores (*r* = 0.30, *P* = 0.045), showing that more contact was made when clients experienced increased depression over the program. All other examinations were statistically nonsignificant (*P* range = 0.53–0.74).

## Discussion

The aim of the present study was to examine the efficacy, acceptability, and feasibility of a previously validated Internet-delivered pain management program, the Pain Course, for adults with chronic pain within the context of a routine practice online therapy clinic. Establishing generalizability of Internet-delivered pain management programs is important prior to wider-scale dissemination. Consistent with past randomized controlled clinical trials^8,11^ supporting the effectiveness of the Pain Course, analyses revealed significant reductions (within-group Cohen’s *d*; average reduction) at posttreatment on primary measures of disability (Cohen’s *d* = 0.45; 18% improvement), depression (Cohen’s *d* = 0.85; 36% improvement), and anxiety (Cohen’s *d* = 0.52; 32% improvement). Furthermore, there were improvements on measures of pain self-efficacy (Cohen’s *d* = 0.83; 29% improvement), pain acceptance (Cohen’s *d* = 0.70; 25% improvement), and fear of movement (Cohen’s *d* = 0.53; 10% improvement). Of importance, observed changes on these measures were maintained at 3-month follow-up. Furthermore, at 3-month follow-up, there was a significant, although small, reduction in pain severity that was not present from baseline to posttreatment (Cohen’s *d* = 0.11; 4% improvement).

When benchmarked against past randomized controlled research trials,^8,11^ the results are consistent, suggesting a high degree of generalizability of findings from research trials of the Pain Course to routine practice. In the largest trial of the Pain Course (*n* = 490), similar improvements were observed for disability (Cohen’s *d* ≥ 0.50; average improvement ≥ 18%), depression (Cohen’s *d* ≥ 0.73; average improvement ≥ 36%), anxiety (Cohen’s *d* ≥ 0.44; average improvement ≥ 32%), pain self-efficacy (Cohen’s *d* ≥ 0.29; average reduction ≥ 15%), pain acceptance (Cohen’s *d* ≥ 0.22; average reduction ≥ 8%), fear of movement (Cohen’s *d* ≥ 0.34; average reduction ≥ 7%), and pain (Cohen’s *d* ≥ .30; average reduction ≥ 12%). Of significance, the outcomes observed from use of the Pain Course in the present study are similar to those reported by other low-intensity face-to-face pain treatment programs.^5,39^

An average of 108.27 min was spent contacting each client. This number was comparable to the level of contact reported in the original Pain Course study (81.54 min).^11^ Of note, the amount of coach time is almost 40 min higher than the average amount of contact time described in a subsequent study of the Pain Course (67.69 min).^8^ One hypothesis is that with increased experience delivering and researching the Pain Course, the developers of the Pain Course have been able to reduce the amount of time required for coaching. In general, given that costs impact scalability, there is value in future research being directed toward understanding the optimal amount of coaching time as well as the optimal training level for coaches (e.g., coach, psychologist, social worker). It is also possible that some clients obtain better outcomes with more contact from a clinician but at this time it remains unknown which clients need a clinician and which clients do not. Despite the coaching time being somewhat higher in our study than described in past studies of the Pain Course, the amount of time required for delivering the Pain Course is very encouraging.

Interestingly, an exploratory analysis of coach contact time and client change scores revealed a statistically significant association for the PHQ-9. Examination of the direction of the correlation indicated that increased coach contact was associated with clients reporting worse outcomes (i.e., increased depression symptoms) at posttreatment. Given that correlational analyses do not specify causal relationships, one interpretation of the data is that coach contact time was responsive and increased during the course of treatment when symptoms were worsening. For example, the coach may have increased contact to assist clients who were demonstrating increased symptom severity at that time. Supporting this interpretation, a similar pattern has been reported between increased therapist contact and increased depressive symptoms when delivering ICBT for depression.^40^

With respect to acceptability of the Pain Course, satisfaction with the program was high, with 73% of clients reporting being either very satisfied or satisfied with treatment, 85% of clients reporting feeling confident recommending the course to others, and 88% agreeing that it was worth their time. Furthermore, completion rates were very high, especially considering that 87% completed the majority of the lessons (e.g., four out of five lessons). Overall, the findings regarding satisfaction compare favorably with those reported in previous trials of the Pain Course.^8,10,11^ In terms of feasibility, from this study, we learned that even in clinical practice with coach support, it takes less than 15 min of contact per week per client to deliver the service. Demonstrating positive outcomes with a coach is important and increases the feasibility of being able to continue to offer the Pain Course in the government-funded Online Therapy Clinic, especially considering that it also only required 30 min of staffing time to screen clients for the service. We also acknowledge, however, that in this study we did not exam total costs associated with delivering the Pain Course. Coaching time is only one cost related to delivering the Pain Course. Other costs include costs associated with the developing and setting up the web platform to deliver the Pain Course and time needed to train and supervise coaches. The current study sets the stage for future research on the cost-effectiveness of the Pain Course. On a positive note, the Pain Course itself was available at no cost.

Taken together, the findings provide evidence that supports further dissemination of the Pain Course within the context of routine clinical care. In particular, the Pain Course may be a first line of contact with clients who may have difficulties attending pain management programs face-to-face due to location, time, mobility, or costs. The improvements of symptoms that were identified are encouraging and highlight the potential of the Pain Course for improving the quality of life of individuals with chronic pain.

Some limitations to the research should be highlighted. First, the sample size was small. A larger sample would allow for examination of moderators of outcomes, such as pain severity or comorbidity or client demographics (e.g., age, gender, education, ethnicity, disability status). Of note, participants in this trial, much like those in past trials of the Pain Course,^8,11^ were predominantly Caucasian, female, middle-aged, married, and university educated. In future trials, it is critical to demonstrate the generalizability of outcomes to other client groups and to explore issues of program reach and recruitment among minority groups. Second, all outcomes were self-reported and we did not collect data on the impact of the Pain Course on medication use or health care utilization. Third, due to limited resources available, we limited the follow-up period to 3 months and were not able to obtain follow-up data on 38% of clients. Having a greater participation rate at follow-up and a longer-term follow-up period would increase confidence in the results.

In terms of other future directions, it would be desirable to systematically explore the benefit of offering the Pain Course as a first step in care (e.g., comparing those who receive the Pain Course versus those who do not in terms of symptom improvement, as well as medication use and health care utilization). It would also be desirable to explore outcomes of the Pain Course in other clinical settings, such as within specialized pain clinics. In terms of additional future directions, it would be instructive to explore whether outcomes could be improved with the support of a multidisciplinary team (e.g., physiotherapy, pharmacy, exercise therapy). As noted in the results, some clients experienced deterioration in outcomes, and being able to step up care for these clients represents an important direction. Of interest, past research shows that the outcomes of the Pain Course are similar whether delivered with weekly clinician contact, client-directed optional contact with a clinician,^8^ or support from a nonclinician.^12^ In order to improve the scalability of the Pain Course, it is important to conduct additional research to better understand the clinical necessity of weekly contact by coaches and the minimum level of training needed to support clients as they complete the Pain Course. It would also be helpful to know the effect of provider type (e.g., social worker, psychologist) and delivery setting (e.g., dedicated e-health unit vs. pain clinic) on client outcomes. Could the Pain Course be delivered with the same level of fidelity and with the same outcomes in a specialized pain clinic that does not specialize in e-health delivery? These are important questions that have implications for the scalability of the Pain Course.

## Conclusions

In summary, in this study, we confirmed the generalizability of past research on the effectiveness of the Pain Course in a provincially funded routine practice online therapy clinic. We identified significant improvements in disability, depression, anxiety, fear of movement, self-efficacy, and pain acceptance. Contributing to confidence in the study findings, we had high course completion and questionnaire completion rates at posttreatment, used measures with strong psychometric properties, and reported effect sizes, percentage improvement, deterioration, client satisfaction, and therapist time to deliver outcomes. The findings add support to offering Internet-delivered pain management programs within routine care as a method of facilitating access to treatment and alleviating the burden of chronic pain.
